# The role of guardians in preventing unintentional injuries among rural children: a mixed-methods study

**DOI:** 10.3389/fpubh.2025.1550541

**Published:** 2025-07-09

**Authors:** Liheng Huang, Dehong Luo

**Affiliations:** ^1^College of Education Sciences, Huaihua University, Huaihua, China; ^2^Wulingshan K-12 Educational Research Center at Huaihua University, Huaihua, China

**Keywords:** rural children, unintentional injury, guardian, countermeasure, injury prevention

## Abstract

**Background:**

Unintentional injuries are a leading public health concern for children, particularly in rural areas of low- and middle-income countries. Guardians are important in injury prevention, yet few studies have systematically examined guardian-related factors in rural areas of China. This study investigates the association between guardian-related factors and unintentional injuries among children in Hunan Province and proposes a three-stage prevention strategy.

**Materials and methods:**

A mixed-methods approach was used. Study I adopted a quantitative design, collecting data through electronic questionnaires from 432 guardians of primary school children across five cities in Hunan Province. Logistic regression and chi-square analyses were performed using statistical software (SPSS) to assess associations between guardian-related factors and unintentional injuries among children. Study II used a qualitative approach, interviewing 30 guardians from 15 counties using a semi-structured guide. Thematic analysis was conducted using NVivo software, followed by Colaizzi’s phenomenological analysis. Findings were synthesized using the Haddon Matrix to develop three-stage preventive measures.

**Findings:**

Study I revealed that guardians who implemented injury prevention measures were less likely to report child injuries (OR = 0.463, *p < 0.05*). However, most guardians lacked consistent preventive actions. Study II identified four key factors contributing to unintentional injuries among children in rural areas: hazardous environmental, inadequate safety education for guardians, children’s behaviors, and lack of regulatory and systemic safety infrastructure in rural areas.

**Conclusion:**

Guardian-related factors significantly influence the prevention of unintentional injuries among rural children. By integrating quantitative and qualitative evidence within the Haddon Matrix model, this study proposes three-stage (pre-, during- and post-injury) preventive measures to reduce risks and enable a theory-driven injury prevention strategy.

## 1 Introduction

The global mortality rate from injuries declined between 2000 and 2019, yet remained high at 57.4 deaths per 100,000 population (UI: 38.7 to 81.4) (1). In China, unintentional injuries such as drowning, falls, traffic accidents, and food poisoning continue to threaten children’s health and development. According to the Chinese National Injury Surveillance System (NISS), 331,663 injury cases were reported among children aged 6 to 17 years from 2015 to 2018 (2).

China’s rapid socio-economic transformation has significantly changed traditional family structures, particularly in rural areas. An increasing number of children now live under intergenerational or single-parent guardianship, which has diminished the educational and supervisory functions of guardians (3). Given that unintentional injuries pose a major threat to children’s development, effective parental and guardian education remains critical for injury prevention (4). Therefore, further research is needed to examine guardians’ educational background and their safety practices in preventing child injuries.

Previous studies suggest that certain characteristics of family guardians, such as education level, number of children under care, and marital status, significantly influence the occurrence of unintentional injuries among children (5). In general, families with highly educated guardians, fewer children, and a strong emphasis on safety education tend to report lower injury rates (6). This may be because less-educated guardians often lack access to injury prevention knowledge, which limits their ability to protect children effectively (7). However, some scholars argue that while education level influences injury prevention awareness, it alone cannot reliably predict a guardian’s overall effectiveness in preventing unintentional injuries (8). So key questions remain: What factors influence the occurrence of unintentional injuries among children? To what extent does guardianship play a role in these incidents?

This study aims to investigate the relationship between the guardianship factors and childhood injuries in rural China. By focusing on guardians in Hunan Province, the research provides a mixed-methods approach to analyze of both quantitative associations and qualitative experiences. The findings aim to provide evidence-based strategies for reducing injury risks and improving child health outcomes.

## 2 Materials and methods

### 2.1 Study design

The study adopted a mixed-methods research approach and was conducted in two phases. Quantitative analysis in Study I: Guardians of elementary school students from five towns in Hunan Province (*n* = 432) were selected for a questionnaire survey. SPSS chi-square tests and logistic regression analysis were used to examine the associations between guardians’ demographic characteristics, supervision behaviors, and other factors with children’s unintentional injuries. Qualitative analysis in Study II: Based on the quantitative findings, 30 guardians with experience of child injuries from 15 counties in Hunan Province were purposively selected for in-depth interviews. NVivo software was employed for qualitative analysis, and Colaizzi’s phenomenological analysis method was applied to identify key factors contributing to unintentional injuries among rural children. Finally, guided by the theoretical framework of the Haddon Model, the quantitative evidence and qualitative results were integrated to propose a three-phase preventive pathway: “pre-event, during-event, and post-event” interventions.

### 2.2 Study I: quantitative analysis

#### 2.2.1 Survey participants

The survey was conducted over an 18-month period from May 2022 to December 2023. Based on the 2021 per capita GDP data published by the website of the People’s Government of Hunan Province (9), the 14 prefecture-level administrative regions were categorized into three economic strata: (1) highly developed regions (per capita GDP > 85,000 yuan, including Changsha-Zhuzhou-Tan region); (2) relatively developed regions (50,000 yuan<per capita GDP < 85,000 yuan, including Hengyang, Yueyang, Yiyang and Changde); and (3) underdeveloped regions (per capita GDP < 50,000 yuan, including Loudi, Shaoyang, Yongzhou, Huaihua, Zhangjiajie and Xiangxi Autonomous Prefecture).

A stratified random sampling approach was used to ensure representativeness across different economic regions. The survey subjects covered the guardians of children from five township-level central primary schools located in both relatively developed and underdeveloped counties. The questionnaire was administered electronically and was distributed in WeChat groups. While this method was time-efficient and convenient, it may have limited the range of respondents and introduced potential selection bias, which is an acknowledged limitation of the study. Detailed information regarding the survey time, school locations, and sample size is provided in Table 1.

**Table 1:** Survey time, school, and sample size for child guardians.

Survey time	School location	Economic development level	School name	Grades of children under guardianship	Sample size
May 2022	Taoyuan County, Changde City, Hunan	Relatively developed	Qihe Town Central Primary School	Grade 1 (2 classes), Grade 4 (2 classes)	164
December 2022	Shaodong County, Shaoyang City, Hunan	Underdeveloped	Shuangjiang Primary School	Grade 5 (2 classes)	45
		Underdeveloped	Yongwon Elementary School	Grade 6 (2 classes)	47
May 2023	Hengnan County, Hengyang City, Hunan	Relatively developed	Huaxing School	Grade 3 (2 classes)	86
December 2023	Zhongfang County, Huaihua City, Hunan	Underdeveloped	Luyang Town Central Primary School	Grades 1 to 3 (3 classes)	90
Total					432

#### 2.2.2 Questionnaire design

The questionnaire was developed based on the World Health Organization’s *Injury Surveillance Guidelines* (Appendix A–O) (10) and adapted to reflect the context of childhood unintentional injuries in rural China.

The questionnaire was compiled based on the occurrence of unintentional injuries among children in rural China. It included the following key areas: 1. Guardian demographics, including guardian type, education level, and number of children under care; 2. Characteristics of the injured child, including age and gender; 3. Guardian knowledge and practices related to injury prevention, including awareness of injury risks, first-aid skills, and provision of safety education.

#### 2.2.3 Quality assurance

To ensure survey quality and consistency, respondents were briefed in advance on the definition of unintentional injury in children. The study adopted the internationally accepted definition of unintentional injury, which refers to external, sudden, unintended, and non-disease-related physical harm (11).

According to the *International Classification of Diseases* (ICD-10), injuries are categorized into 12 types, including falls, sharp-object injuries, burns, traffic accidents, animal bites, drowning, and poisoning (12).

Inclusion criteria for reported injuries were: 1. The child sustained an injury due to an unintentional incident and received medical attention or first aid either at home or in a healthcare facility; 2. The child was unable to engage in normal activities for at least one full day due to the injury. Cases meeting either criterion were classified as unintentional injuries (13).

A total of 432 questionnaires were collected. Responses completed in less than 60 s (indicating careless responses) or more than 6,000 s (suggesting prolonged interruptions) were excluded. A final sample of 419 valid responses was included in statistical analysis.

#### 2.2.4 Statistical analysis

Survey data were initially organized in Microsoft Excel and subsequently analyzed using SPSS version 26.0. The analysis proceeded in three stages: Firstly, descriptive analysis was conducted on the basic characteristics of guardians and the occurrence of unintentional injuries among children. Second, statistical significance tests were performed to examine associations between individual guardian characteristics and the incidence of child injuries. Finally, a logistic regression analysis was carried out, using the occurrence of unintentional injuries among rural children as the dependent variables, and guardians’ characteristics and their education and guardianship practices as the independent variables.

### 2.3 Study II: qualitative analysis

While quantitative research is useful for measuring and analyzing phenomena through objective data, it often lacks contextual sensitivity and may not fully capture the complexity of certain issues across different settings. In contrast, qualitative research methods allow for deeper, more comprehensive exploration of underlying phenomena related to the research questions. Therefore, Study II adopts a qualitative approach to investigate: what are the key factors contributing to unintentional injuries among rural children? And what is the specific role and influence of guardians in this process?

This study used in-depth interviews with guardians of children in rural areas to gather detailed case information about unintentional injuries. Data was analyzed using NVivo15.0 software for coding and theme identification. Then Colaizzi’s seven-step analysis method was used to explore the context under which these injuries occurred and to understand guardians’ perspectives and emotional responses on children’s unintentional injuries. as the aim was to identify the key factors contributing to children’s unintentional injuries in rural areas and, subsequently, to propose targeted prevention strategies using the Haddon Matrix.

The seven steps of Colaizzi’s analysis method include: 1. Repeated reading and familiarization with the interview transcripts; 2. Extracting statements and narratives relevant to the research objectives; 3. Coding recurring views and sentiments; 4. Organizing meaningful units and clustering them into preliminary themes; 5. Formulating detailed theme descriptions; 6. Refining and synthesizing themes into concise, high-density thematic structures; 7. Validating the derived themes by returning to participants to confirm that the analysis accurately reflects their lived experiences (14).

The Haddon Matrix is a classical theoretical model in injury prevention research. It conceptualizes injury events as resulting from the interaction of three key elements: the host (individual), the agent and the environment (both physical and social). These factors operate three temporal phases: pre-event, event, and post-event (15). By organizing risk factors into a clear matrix, the model provides a structured way to identify and classify preventive strategies before, during, and after an injury event (16). Its visual clarity and analytic depth support the development of creative and evidence-based prevention measures, including for child injury contexts (17).

#### 2.3.1 Interview participants

Using purposive sampling, this study invited 30 guardians whose children had experienced at least one unintentional injury within the past 3 years. Participants were selected from 15 counties in Hunan Province: Taoyuan County and Hanshou County (Changde City); Hengnan County (Hengyang City); Shaodong County (Shaodong City); Zhongfang County, Yuanling County, Zhijiang County (Huaihua City); Dao County, Ningyuan County, Jianghua Yao Autonomous County (Yongzhou City); Yanling County (Zhuzhou City); Lianyuan County (Loudi City); Xiangyin County (Yueyang City); Taojiang County (Yiyang City); Fenghuang County (Jishou City).

Among these, 10 participants were from the original survey respondents in Study I, while the other 20 participants were introduced through referrals from trusted contacts familiar with rural guardians. Each interviewee was assigned a code based on the format: “ID code-child’s gender (B/G)-guardian type.” Participant demographics and details are summarized in Table 2.

**Table 2:** Details of interviewee profiles.

Code	Guardian type	Guardian education level	Child’s gender
1-B-FQMQ	Father and mother	Junior high school	Boy
2-B-FQMQ	Father and mother	Junior high school	Boy
3-G-FQMQ	Father and mother	Junior high school	Girl
4-B-FQMQ	Father and mother	Junior high school	Boy
5-B-FQMQ	Father and mother	Elementary school	Boy
6-G-FQMQ	Father and mother	Junior high school	Girl
7-G-FQMQ	Father and mother	High school	Girl
8-G-FQMQ	Father and mother	High school	Girl
9-B-FQMQ	Father and mother	Junior high school	Boy
10-G-FQMQ	Father and mother	Junior high school	Girl
11-G-FQMQ	Father and mother	Elementary school	Girl
12-G-FQMQ	Father and mother	Junior high school	Girl
13-B-FQMQ	Father and mother	Elementary school	Boy
14-G-FQMQ	Father and mother	Elementary school	Girl
15-G-FQ	Father	Elementary school	Girl
16-G-MQ	Mother	Junior high school	Girl
17-B-MQ	Mother	High school	Boy
18-G-MQ	Mother	Junior high school	Girl
19-G-YYNN	Grandparents	Elementary school	Girl
20-G-YYNN	Grandparents	Elementary school	Girl
21-G-YYNN	Grandparents	Junior high school	Girl
22-B-YYNN	Grandparents	Junior high school	Boy
23-B-YYNN	Grandparents	Junior high school	Boy
24-G-YYNN	Grandparents	Junior high school	Girl
25-B-YYNN	Grandparents	High school	Boy
26-B-WGWP	Maternal grandparents	Elementary school	Boy
27-G-WGWP	Maternal grandparents	High school	Girl
28-G-WGWP	Maternal grandparents	Junior high school	Girl
29-B-WGWP	Maternal grandparents	Elementary school	Boy
30-B-WGWP	Maternal grandparents	Elementary school	Boy

#### 2.3.2 Interview questions

Based on a thorough literature review, group discussions among project members, and consultation with field experts, a semi-structured interview guide was developed to align with the study objectives. The interview focused primarily on two areas: 1. guardians’ practices and perceptions regarding injury prevention, education, and response; and 2. contextual details of rural child injury cases.

The interview outline included questions such as:

1. Guardians’ practices and perceptions regarding injury prevention, education, and response:

“Are you the primary guardian for the child?”
“What is your relationship to the child?”
“Do you worry about your child experiencing an injury?”
“Do you believe childhood injuries are preventable?”
“Have you taken any specific actions to prevent unintentional injuries in children?”
“Do you have any knowledge or skills in handling child injuries?”
“Can you describe how you supervise and educate your child regarding safety at home?”
The specific questions about the occurrence of unintentional injuries for children in rural areas:

“What type of injury did your child experience?”
“Can you describe how the injury occurred?”
“What specific factors contributed to the injury?”
“Were you nearby when the incident happened?”
“How did people around you respond?”
“In your opinion, what are the main risk factors for child injury?”
“What do you think are the most effective ways to prevent child injury?”
“Have you received any safety education from local schools or government authorities (county, township, village levels)? How effective was it in helping you prevent injuries?”

#### 2.3.3 Quality control

The diagnostic criteria for unintentional injuries among children were consistent with those used in Study I. All interviewers received training in qualitative interview techniques to ensure consistency and avoid leading or suggestive questioning.

Prior to each interview, the researcher explained the study purpose and obtained verbal informed consent from each participant. Interviews were conducted remotely by phone, at times convenient to the participants, and lasted approximately 30 to 60 min.

While most interviews adhered closely to the established guide, minor adjustments were made based on individual cases, particularly to gather more detailed insights on specific injury incidents. Each interview was audio recorded with permission, and notes were taken to capture key points and contextual details.

#### 2.3.4 Data processing and analysis

Interview transcripts were prepared within 24 h of each session. Transcripts were cross verified using audio recordings and interviewer notes to ensure accuracy and completeness.

Data was analyzed using NVivo 15.0 software and Colaizzi’s seven-step phenomenological method. This dual approach enabled both thematic extraction and interpretive depth. The analysis aimed to understand guardians’ thoughts, beliefs, and emotional responses related to child injury; the underlying social and environmental factors contributing to injury events; and the hidden meanings within guardians’ narratives.

Thematic results were then interpreted using the Haddon Matrix framework to categorize risk factors across the pre-event, event, and post-event stages (16). This analytical approach provided a foundation for evidence-based policy recommendations and the design of targeted intervention strategies for preventing unintentional injuries among rural children.

## 3 Findings

### 3.1 Study I: quantitative results

#### 3.1.1 Descriptive statistical analysis of guardians

Table 3 shows the results of descriptive statistical analysis for the guardian characteristics.

**Table 3:** Results of descriptive statistical analysis of guardians (*n = 419*).

Item	Number of respondents	Percentage	Whether there was any accident happened to their children	
			No (*n = 185*)	Yes (*n = 234*)
Type of guardian				
Father and mother	297	70.9	130	167
Father	12	2.9	6	6
Mother	55	13.1	25	30
Grandparents	33	7.9	14	19
Grandparents	14	3.3	4	10
other	8	1.9	6	2
The number of children under guardian care				
1	110	26.3	50	60
2	240	57.3	106	134
3	59	14.1	26	33
4 or more	10	2.4	3	7
The education level of the guardian				
Elementary school	71	16.9	29	42
Junior high school	170	40.6	75	95
High school	114	27.2	48	66
University	64	15.3	33	31

Among the 419 guardians surveyed, 234 (55.84%) reported that the children under their care had experienced at least one unintentional injury. Most guardians were biological parents (70.9%), followed by grandparents and others.

The number of children under each guardian’s care was typically two (57.3%). In terms of the education level, 40.6% of guardians completed junior high school, 27.2% completed senior high school, and 15.3% hold a university degree.

#### 3.1.2 Bivariate analysis of guardian-related factors and children’s unintentional injuries

Table 4 shows the results of chi-square test, using the occurrence of unintentional injuries among rural children as the dependent variable. Two guardian-related variables showed statistically significant associations with injury occurrence (*p* < 0.05): whether the guardians had taken preventive measures against unintentional injuries, and whether they had mastered first aid skills for household injuries.

**Table 4:** Chi-square test results for guardianship factors and the occurrence of unintentional injuries of children.

Variable	Pearson Chi-square value	Degree of freedom	*p*-value
Type of guardian	4.727	5	0.457
Number of children under guardianship	0.888	3	0.841
Guardian’s education level	1.934	3	0.590
Presence of guardian during injury occurrence	0.158	1	0.695
Has taken preventive measures against child injury	8.154	1	*P < 0.05*
Concern about child experiencing injury	4.904	4	0.276
Has received training in first aid for unintentional injury	0.003	1	0.957
Has mastered basic first aid techniques	6.790	2	*P < 0.05*
Availability of basic first aid supplies at home	3.163	4	0.535
Has provided safety education to the child	3.027	4	0.553

As shown in Table 5, a binary logistic regression was conducted with unintentional injuries of rural children (no injuries = 0, injuries = 1) as the dependent variable. Independent variables included the type of guardian, educational background, number of children under guardianship, and specific guardianship practices related to injury prevention and first aid education.

**Table 5:** Logistic regression analysis of guardian-related factors associated with unintentional injury in children.

Variable	B	Standard error	Wald value	*p*-value	OR	95%CI
Type of guardian	1.716	0.883	3.777	0.052	5.561	0.985 ~ 31.376
Number of children under guardianship	−0.526	0.808	0.424	0.515	0.591	0.121 ~ 2.879
Guardian’s education level	0.38	0.415	0.839	0.36	1.462	0.649 ~ 3.297
Presence of guardian during injury occurrence	0.134	0.223	0.361	0.548	1.143	0.739 ~ 1.769
Has implemented preventive measures for child injury	−0.771	0.28	7.557	*P < 0.05*	0.463	0.267 ~ 0.802
Concerned about potential child injury	−0.377	0.252	2.227	0.136	0.686	0.418 ~ 1.125
Has received household first aid training	0.056	0.251	0.05	0.824	1.057	0.647 ~ 1.728
Has mastered emergency first aid skills	0.541	0.253	4.558	*P < 0.05*	1.718	1.045 ~ 2.823
Availability of basic first aid supplies at home	0.485	1.04	0.218	0.641	1.624	0.212 ~ 12.46
Has provided safety education to child	0.293	0.316	0.864	0.352	1.341	0.722 ~ 2.489

The results indicate that two variables were statistically significant predictors of injury occurrence: whether the guardian implemented injury prevention measures and whether the guardian mastered first aid skills (*p < 0.05*). Other variables such as guardian type, number of children under care, and the education level of guardians were not significantly associated with the likelihood of injury.

Our findings indicate that while guardian-related factors contribute to children’s unintentional injury risks, only selected factors show significant statistically significant effects. The development of children is inherently uncertain, with injury risk being shaped not only by guardians’ education and safety practices, but also by environmental hazards and children’s own risk awareness (18). The quantitative study (Study I) alone proved insufficient for comprehensively examining these factors or determining the extent of the impact by guardian-related factors on unintentional injuries among rural children.

To address these limitations, we implemented a complementary qualitative investigation (Study II). The follow-up study used in-depth interviews with guardians, with subsequent data analysis using NVivo15.0 software and Colaizzi’s seven-step analysis method. This approach enabled deeper exploration of the multifaceted factors influencing unintentional injuries among rural children.

### 3.2 Study II: qualitative results

**Figure 1:**
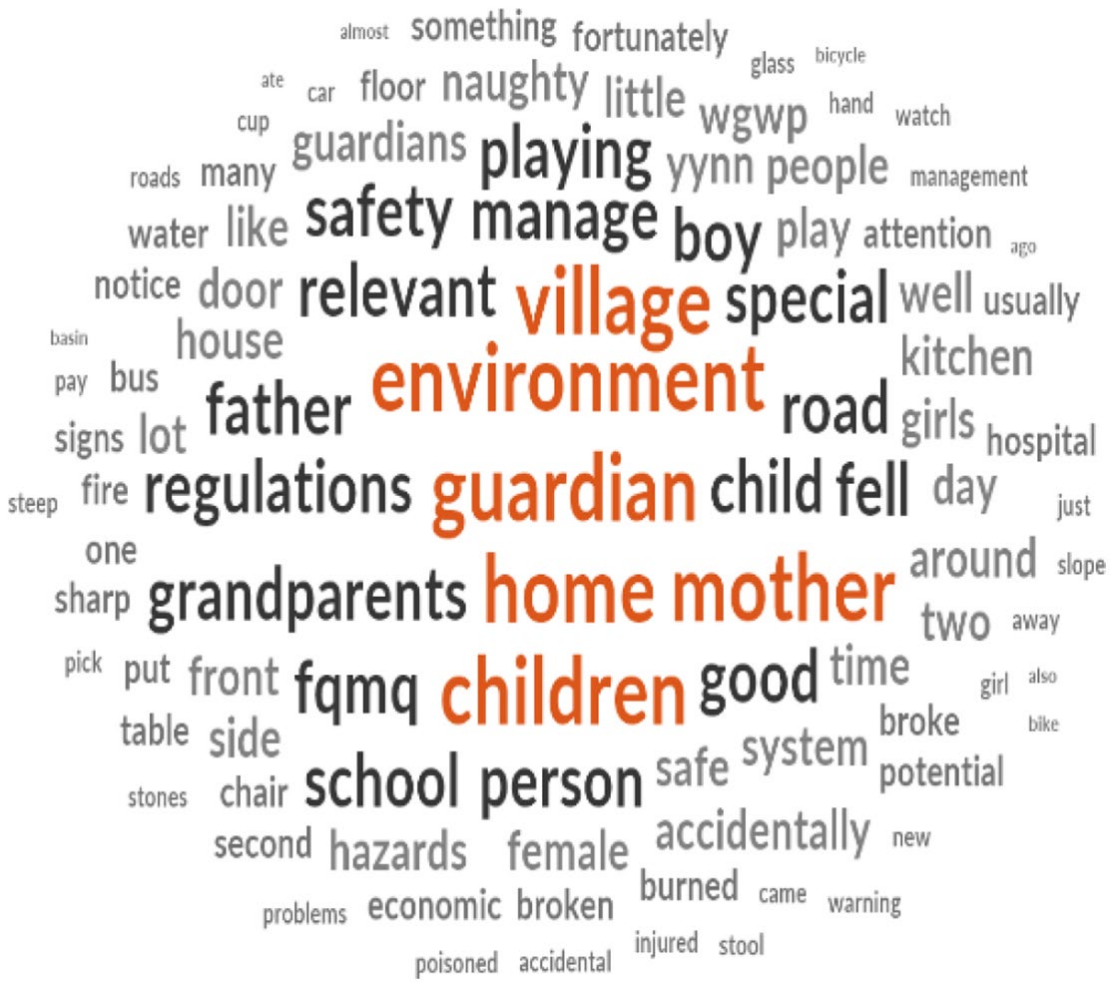
Word cloud of interview transcripts on the unintentional injuries among rural children.

**Figure 2:**
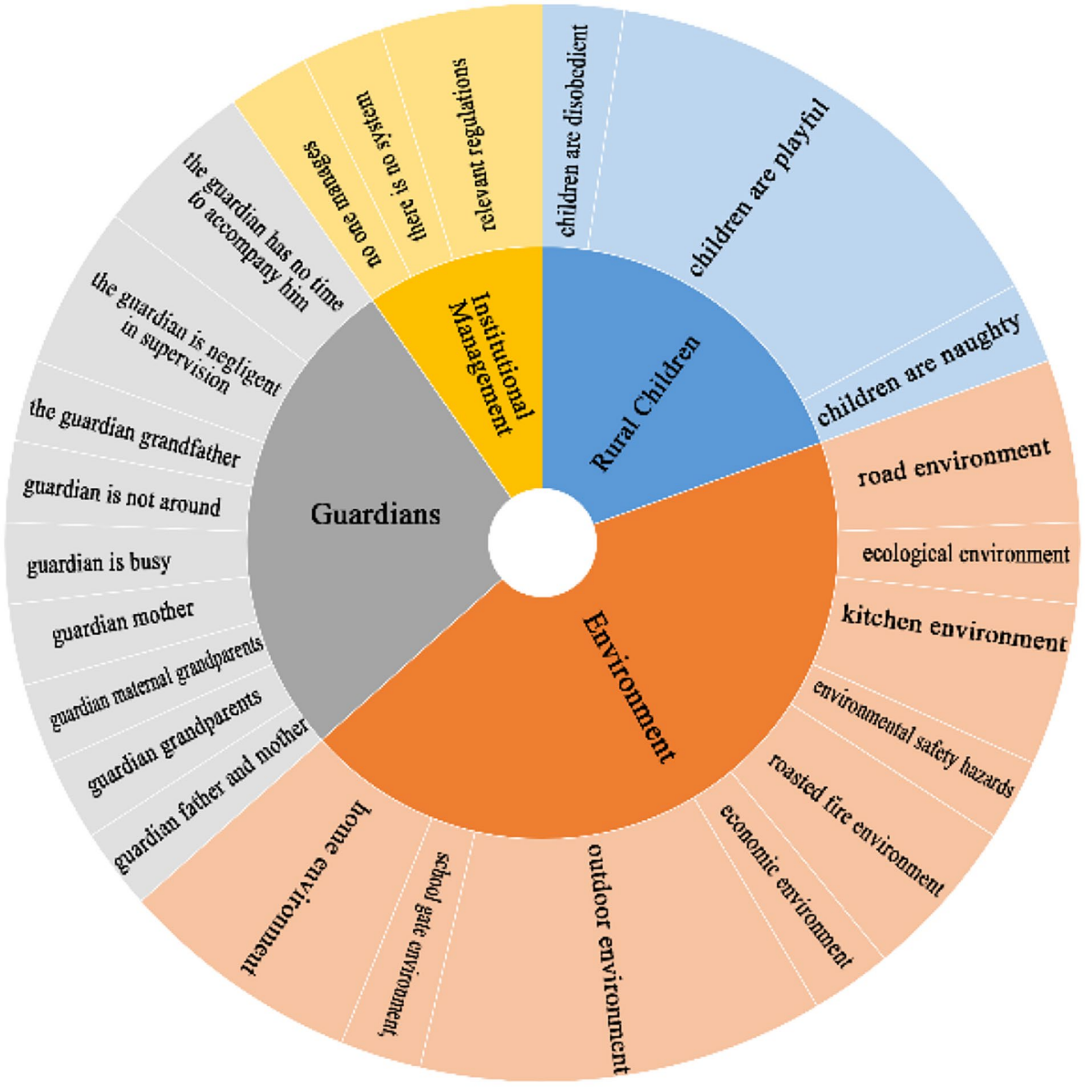
Structural model of influencing factors resulting in unintentional injuries among rural children.

The interview transcripts were analyzed using NVivo15.0 software to generate word frequency visualizations (word cloud, Figure 1). Following automated coding by NVivo, thematic analysis identified 24 sub-themes (second-level codes) into 4 primary thematic categories (first-level themes) nodes: 1. environmental factors, 2. guardian-related factors, 3. child-related factors, and 4. institutional/ regulatory factors. These four thematic domains represent the key influences on the occurrence of unintentional injury risk among rural children. The structure of these influencing factors is shown in Figure 2.

Among the four categories, guardian-related factors ranked second. This indicates that while guardians play an important role, environmental hazards emerge as the dominant contributor to child injuries. Environmental risks included exposure to unsafe outdoor rural settings, unprotected spaces around homes and schools, and unsafe living conditions within the home.

While NVivo software provided preliminary automatic coding and topic identification, its semantic interpretation capabilities were limited in depth. Therefore, manual refinement of the analysis was carried out. Based on research design, team members first conducted trial coding on selected transcripts and held collaborative discussions to resolve inconsistencies and interpretation challenges. Once consensus was reached, full coding of all transcripts was conducted, followed by inductive analysis and thematic synthesis.

Through repeated reading and interpretation of participants’ narratives, meaningful statements were identified, organized into semantic units, and categorized. This process resulted in four core themes representing the main influences on unintentional injuries among rural children: environmental factors, guardian-related factors, child-related factors, institutional and regulatory factors, as shown in Table 6.

**Table 6:** Key themes and quotes from interviews with guardians of rural children.

Theme	Subtheme	Meaningful units	Examples scenario/Quote
Environment	Unsafe home and rural environments; Complex, unregulated rural terrain; Flooded and unprotected surroundings	Climb a highchair in the house; There are many children waiting on the side of the road; Country roads are steep and narrow in some places; When it rains, the road is very slippery;	“He climbed a highchair at home to fetch something. The chair tipped over, and he fell and broke his teeth” (1-B-FQMQ); “There are many children waiting for buses by the roadside” (3-G-FQMQ); “Some rural roads are steep, some narrow, and very slippery when it rains” (12-G-FQMQ); “There was a ditch in front of our house. It rained heavily that day, and the ditch overflowed. We did not notice that he had walked there” (23-B-YYNN).
Guardian	Guardian distraction or absence; Neglect; No time for school pickup	Not around the child; As soon as I turned around to get something; I did not pay attention; There is no time to pick up children from school;	“We did not notice he climbed up the chair. The chair tipped over, and he fell and broke his teeth” (1-B-FQMQ); “I turned around to get something, and my child burned their hand near the charcoal fire” (24-G-YYNN); “In the blink of an eye, without noticing, the child broke the water glass, and the splinter poked him in the forehead” (17-B-MQ); “We did not have time to pick him up. He rode his bike home alone, slipped in the rain, and fell” (25-B-YYNN).
Children	Playfulness, curiosity, lack of risk awareness; Impulsiveness and lack of consequence awareness	The child is very naughty, climbing up and down at home; Stand on a chair and cook noodles; After watching the cartoon, I thought people could fly, and I wanted to show it.	“The child is very playful, always climbing up and down at home. The chair tipped over” (1-B-FQMQ); “The child likes to explore new things. He was standing on a chair to cook noodles. That time He tried to cook noodles while standing on a chair, but lost balance and burned his hand” (2-B-FQMQ); “The child does not what is dangerous. He watched a cartoon where people could fly and tried to show his sister by jumping from the second floor” (10-G-FQMQ);
Institutional and regulatory management	Lack of road signage and public supervision; Unsafe traffic and poor vehicle control; The roads, rivers and wells have maintenance issues	There is no administrator at the bus stop; There are no signs where you are waiting; There are no fence or barriers around the well; The road is in front of the house.	“There were no warning signs or barriers near the well. He slipped on a rainy day and fell in” (22-B-YYNN); “He was playing in front of the house when a car hit him. The driver did not have adequate driving skills” (13-B-FQMQ); “He secretly used the trolley. One time he lost control and rolled down a slope with it” (27-G-WGWP).

While rigorous efforts were made to ensure coding accuracy, human error and interpretive bias remain possible, including potential omissions or misinterpretations. The sample was intentionally diverse, including different types of guardians and various injury scenarios. After the primary themes were saturated, follow-up interviews with additional guardians did not yield new themes, suggesting a degree of thematic saturation. However, further validation in future studies is needed to check the robustness of the findings.

## 4 Discussion

### 4.1 Descriptive analysis of guardians of rural children with unintentional injuries

As shown in Table 3, descriptive statistics reveal the basic characteristics of rural children’s guardians, including the type of guardianship, the number of children under guardianship, and the education level of guardians. These results are largely consistent with previous studies (19).

Regarding the types of guardianship, the most common arrangements were dual-parent guardianship (both mother and father), followed by mother and grandparent guardianship. In single-parent families, guardianship was most often undertaken by the mother (13). A notable portion of cases involved “other” types of guardianship, which included uncles, aunts, older siblings, or even child self-care. In certain rural households, especially where grandparents have passed away and parents are migrant workers in urban areas, children are left in the care of extended family or must self-supervise.

For the number of children under guardianship, most guardians were responsible for two children. However, in households with grandparent guardianship, it was common for guardians to care for three or four children simultaneously. This reflects traditional extended family structures, where grandparents assume responsibility for multiple grandchildren.

In terms of the education level, most guardians had completed junior high school, although a notable minority had attained university-level education. This may be partly attributed to national policy initiatives such as the implementation of the *Special Post Teachers Programme* launched in 2006, and the broader *Rural Revitalization Strategy*, which has encouraged university graduates to take up employment in township-level positions. As a result, the overall educational level among some rural populations has improved. Nevertheless, the data suggests that higher education levels among guardians did not translate into a significant reduction in child injury risk, which is consistent with the findings of relevant scholars (8).

### 4.2 Analysis of guardian-related factors for unintentional injuries among rural children

As shown in Tables 3, 4, two guardian-related factors were significantly associated with the occurrence of unintentional injuries among rural children: 1. whether the guardian had taken injury prevention measures, and 2. whether the guardian has mastered first-aid skills.

Children whose guardians had not taken any accident prevention measures were more likely to experience unintentional injuries compared to those whose guardians had taken such actions (OR = 0.463, 95% CI [0.267, 0.802], *p < 0.05*). Similarly, children whose guardians lacked first-aid skills had a higher probability of injury than those whose guardians had acquired such skills (OR = 1.718, 95% CI [1.045, 2.823], *p < 0.05*).

The finding that Exp(B) < 1 for injury prevention measures suggests a negative correlation between this variable and injury occurrence. That means with each increase in preventive behavior, the likelihood of child injury decreases. In contrast, Exp(B) > 1 for first-aid skills indicates a positive association, it may reflect greater injury awareness or recall among more knowledgeable guardians, rather than a direct causal relationship. This highlights that while first-aid proficiency is crucial for injury mitigation, it does not necessarily reduce injury incidence itself.

Other variables, including guardian type, number of children under guardianship, and education level of guardians, did not exhibit statistically significant associations with injury occurrence. Similarly, variables such as whether the guardian was present at the time of injury, their level of concern about potential injury, and their provision of safety education also showed no significant effects.

The findings highlight the importance of pre-injury prevention. Guardians who proactively implement safety measures can substantially reduce the likelihood of child injuries. This aligns with previous studies (20). On the contrary, if the guardian does not pay attention to the preventive measures of unintentional injury of children and does not eliminate the existence of unintentional injury factors in time, children are very prone to unintentional injuries. For example, if a guardian prohibits a child from swimming alone in unsupervised or unsafe areas, or ensures accompaniment to a safe swimming location, the risk of drowning can be significantly reduced. Also, if a child unfortunately experiences a burn or near-drowning incident, a guardian trained in emergency response may help minimize the severity of the injury.

The factors influencing unintentional injuries among children in rural areas are multifaceted. Previous research using univariate analysis has identified several relevant variables, including: the child’s gender, age and personality; parents’ education level and occupation; and whether the child is a “left-behind” child; frequency of Internet use; whether the guardian is a biological parent’ guardian’s age; the level of harmony in the parents’ relationship; whether the father or the mother is working away from home; whether the household stores fireworks or explosives; and whether physical fights frequently occur in the surrounding area, including the child’s own participation in fights (21). In a follow-up multivariate logistic regression analysis that uses the statistically significant variables from the univariate screening as independent variables and the occurrence of child injury as the dependent variable, the results showed that being male, being a “left-behind” child, household storage of fireworks, and frequent neighborhood violence significantly increased the risk of unintentional injuries among children. Other variables did not show significant associations (21). These significant influencing factors can be broadly categorized into three groups: 1. child-related factors; 2. family-level factors; and 3. environmental factors (22).

### 4.3 The role of guardians in preventing unintentional injuries

#### 4.3.1 Guardians struggle to cope with the complexity of the rural environments

Findings from Study II indicate that rural environment is the most significant contributors to the occurrence of unintentional injuries among rural children. All interview cases involved environmental influences, especially from the complex and unpredictable nature of outdoor rural settings, as well as hidden safety hazards in the home environment.

Despite their concern for safety, guardians often lack the time, energy, or capacity to effectively manage the diverse risks present both indoors and outdoors. For instance, “On a rainy day, the small ditch near the house overflowed into a stream. The child went outside alone and accidentally drowned in the water” (23-B-YYNN). “There is a steep slope in front of our house. The child accidentally slipped and fell and injured himself” (7-G-FQMQ). “The child was playing barefoot outside and got his foot cut by broken glass pieces” (29-B-WGWP).

Indoor environments also posed risks. In some cases, injuries occurred due to inadequate safety infrastructure, such as beds without guardrail. “I’m quite strict with my child, so injuries have been rare. But one time, she was playing with her brother on the bed and fell off” (5-B-FQMQ). “He climbed up a very highchair to fetch something. The chair tipped over, and he fell and broke his teeth” (1-B-FQMQ).

These findings suggest that geographic and environmental factors are deeply embedded in the injury risk profile for rural children, Guardians must recognize and address the unique environmental challenges posed by rural settings. Consistent with earlier research (23), this study highlights the need for environment-focused prevention strategies alongside guardian supervision.

#### 4.3.2 Guardian-related factors alone were insufficient to prevent injuries

Out of the total cases, 26 incidents were directly linked to guardian-related factors. Interviews showed that while guardians were generally aware of the risks and concerned about their children’s safety, many lacked concrete preventive actions and sufficient knowledge to intervene effectively.

Common themes included neglect, lack of hazard awareness and knowledge gaps. “I usually do not pay much attention to what might hurt the child at home” (17-B-MQ); “One time I forgot to put away the scissors. The child started playing with them and accidentally poked himself” (18-G-MQ); “I did not notice the child had wandered to the edge of a dangerous road to play” (13-B-FQMQ); “I did not realize the bicycle brakes were broken. The child went downhill, could not stop, and fractured a bone” (16-G-MQ); “I did not know the mushrooms I picked were poisonous. The child ate them and was poisoned” (14-G-FQMQ, 20-G-YYNN).

These examples show that guardians face challenges in providing adequate safety education and guardianship, largely due to limited knowledge, time constraints, and insufficient emergency preparedness.

Findings from Study I reinforce this point where actual preventive actions, not merely awareness, are the most critical factor in reducing injury risk. Guardians must be empowered not only to recognize dangers but also to respond proactively. Improving their knowledge base, vigilance, and daily monitoring practices is essential for improving child safety outcomes in rural areas.

#### 4.3.3 Guardians feel incapable of supervising children’s psychology and behavior

In 22 of the interview cases, child-related factors were identified as the primary contributors to unintentional injuries. These included high levels of physical activity, playfulness, curiosity, poor risk perception, and an inability to respond appropriately to dangerous situations. Although guardians expressed concern and attempted to supervise their children, many felt overwhelmed and unable to manage their behavior effectively due to time and energy constraints.

Several injury cases showed this dynamic: a child climbed a high chair to fetch something and broke his front teeth (1-B-FQMQ); another tried to grab a glass of water, broke it, and was cut on the forehead by the shards (17-B-MQ); one child attempted to imitate a cartoon character by jumping from the second floor and was seriously injured (10-G-FQMQ); another secretly took the family’s trolly and lost control on a downhill slope, resulting in injury (27-G-WGWP); In one case, a young child’s clothing was caught in a vehicle door, but the child failed to alert the driver in time due to their age and inability to respond effectively (3-G-FQMQ).

The interviewed guardians expressed their frustration and helplessness, stating they lacked the capacity to both manage the household and fully supervise their children’s risky behaviors. This finding is consistent with Study I, which found that guardians’ safety education efforts alone did not have a statistically significant impact on the likelihood of child injury.

#### 4.3.4 Guardians overlook the importance of safety regulations and management

In 18 cases of the unintentional injuries, the absence of institutional safety regulations and management systems was identified as a major contributing factor. Despite expressing concern about injuries, most guardians viewed these events as unavoidable and rarely acknowledged the role that schools, local governments, or village committees could play in injury prevention.

Existing research has emphasized the importance of implementing school-route-focused traffic safety management systems to prevent unintentional injuries among children (24). This is particularly relevant as many injury incidents occur near schools and village communities. For example, “Children often board vehicles near schools and community intersections, where there is no supervision or safety infrastructure. That’s when accidents happen” (3-G-FQMQ). “There are no adults monitoring public areas in the village. Kids climb trees and walls freely, and there is no protective infrastructure” (4-B-FQMQ). “There are no safety guidelines or equipment when using open charcoal fires in rural homes. My child got burned while warming themselves” (24-G-YYNN). “Dog ownership is not regulated in our village, and my child was bitten by a dog” (15-G-FQ, 28-G-WGWP).

The issue is exacerbated by gaps in rural infrastructure. While China’s Rural Revitalization Policy has led to widespread road development, many newly constructed homes are located directly beside roads. “However, speed controls, warning signs, traffic lights, and pedestrian crossings are often absent, increasing the risk of traffic-related injuries for children” (13-B-FQMQ).

In order to save costs in rural construction, rural housing often lacks proper safety features, such as stair railings and slope barriers. This has resulted in multiple cases where children fell from stairs or elevated platforms (9-B-FQMQ, 21-G-YYNN).

These findings indicate that many institutions, including schools, local governments, and community organizations, lack adequate safety regulations, enforcement mechanisms, and child protection infrastructure. Moreover, guardians often fail to recognize the importance of engaging with these institutions to collaboratively prevent injuries.

## 5 Summary and recommendations

### 5.1 Pre-injury phase: enhancing guardian supervision and safety education

In the pre-injury phase, guardians should adopt proactive hazard mitigation and improved supervision practices. Findings from Study I highlight that consistent prevention measures significantly reduce the possibility of unintentional incidents. Inadequate or improper home supervision often leads to preventable injuries (25).

This study revealed that rural guardians often follow a “hands-off” or laissez-faire parenting approach, allowing children to roam freely in outdoor environments with minimal supervision. For example, in one case, a mother did not accompany her child while cycling and failed to notice malfunctioning brakes, resulting in a serious injury. Also, some children experience long-term absence of their legal guardians, typically due to labor migration, financial pressure, or divorce. In such cases, children are deprived of both supervision and the opportunity to learn essential self-protection skills from their parents.

Guardians, especially parents, should prioritize increased engagement and companionship with children to reduce instances of unsupervised outdoor activities. However, overprotection and complete confinement at home are not ideal either. Research shows that excessive restriction can increase family tension and ironically reduce effective supervision, potentially raising injury risk (26).

To create a healthy and safe environment, guardians should identify and mitigate household hazards, adopt injury prevention measures (27), and provide children with continuous safety guidance. Given children’s natural tendency toward exploration and risk-taking, some injury events may be unpredictable. Nevertheless, with targeted and comprehensive interventions, guardians can play an important role in reducing unintentional injuries risks (17). For example, simple actions such as constant attention to children during playtime; immediate response when the child is approached by unfamiliar individuals; educating children to avoid dangerous areas such as roads, rivers, and ponds; reminding children to hold an adult’s hand when crossing the street; keeping children within sight when outdoors and instructing them to inform adults of any issues.

On the other hand, guardians must raise children’s self-protection awareness and risk prevention ability through daily safety education. For example, children should be closely monitored when exposed to potential hazards and properly guided through those situations to ensure safety.

Children are inherently curious and often lack the ability to distinguish between safe and dangerous behaviors. They are eager to try new things, but their limited social experience puts them at high risk. Guardians should consistently teach children about the hidden dangers in everyday situations, share basic safety knowledge, and help them understand the consequences of risky behavior. This can be achieved through practical guidance, such as do not eat unfamiliar food, avoid unsupervised water play, never handle knives or fire, wear helmets when cycling, and refrain from horseplay near traffic. They can also use videos to demonstrate common injury types and their consequences; ongoing verbal instruction and reminders; and clear rules about what is and is not acceptable behavior.

Only through consistent and structured safety education can children learn safety knowledge, develop protective behaviors, and enhance their ability to avoid injury. Increasing safety awareness not only promotes children’s understanding of personal risk but also builds their capacity to respond to and prevent injury events more effectively.

### 5.2 During-injury phase: improving guardian response capabilities

When an injury occurs or is about to occur, guardians must demonstrate prompt and effective intervention to mitigate harm Our interview data suggest that while guardians often recognize the occurrence of injury, their emergency response knowledge is limited. For example, some guardians reported using cold water to treat burns but were unclear about appropriate medications or further treatment. One interviewee stated: “The child had vomiting and diarrhea, and we took them to the hospital. It turned out they had eaten spoiled watermelon and had food poisoning” (20-G-YYNN).

This highlights a critical gap: although guardians value child safety, many lack practical skills and timely intervention strategies, resulting in delayed responses or ineffective treatment. As a result, the likelihood and severity of unintentional injuries among rural children remain high. There is a clear need to improve guardians’ capacity for real-time injury response.

Previous research confirms that the incidence of injury among rural children is closely related to parental awareness and preparedness. “Effective injury prevention in rural settings depends primarily on home-based interventions, and improving guardian knowledge, attitudes, and practices is essential to changing injury-related caregiving behaviors” (5). Guardians should master some basic safety knowledge and first-aid methods, such as emergency medical response methods, including the Heimlich manoeuvre for foreign-body airway obstruction and cardiopulmonary resuscitation (CPR) in cases of drowning or electric shock.

### 5.3 Post-injury phase: multiple-stakeholder collaboration for effective treatment and prevention

After an injury has occurred, the guardian should immediately seek medical care for the child, properly handle the injury, and adopt measures to prevent recurrence. Prior research indicates that in many cases of childhood injury, guardians either handle the situation on their own or do not treat it at all. This behavior is often linked to a lack of awareness about appropriate injury management, financial constraints in rural households, and limited attention given to injury consequences (28).

Interviews from this study revealed that most guardians attempted to respond to injuries, but the quality and the degree of treatment were often insufficient. For example, one guardian reported simply bandaging and applying ointment after a fall injury, without conducting diagnostic imaging (such as an ultrasound or X-ray) to assess for fractures (16-G-MQ).

Given that unintentional injuries among rural children frequently occur near schools and village communities, it is essential that guardians cooperate with government bodies, schools, and civil society organizations to improve response and prevention systems. The healthy and safe growth of children is a continuous and shared responsibility. While biological parents are the legal guardians, in cases where they are unavailable, due to labor migration, health issues, or divorce, primary guardianship responsibilities should be assumed by close relatives or, where necessary, delegated to appropriate administrative bodies, such as residents’ committees, village committees, or departments of civil affairs (29).

In situations where legal guardians formally entrust guardianship to others, all parties including the parent, the guardian, and the child should maintain regular communication (29). It is the legal guardian’s duty to remain actively involved in remote supervision, ensuring not only emotional connection but also consistent monitoring of the child’s academic performance, emotional well-being, and personal safety. Casual greetings are not sufficient, and there must be ongoing engagement in the child’s development and safety (30).

In addition, prevention efforts must not be neglected. Guardians, especially grandparents, should participate in community and school-organized safety education programs to improve their knowledge about safety. Collaborative efforts among schools, local governments, and community organizations are needed to systematically identify and eliminate environmental hazards around school zones and village areas. These efforts should include the development and enforcement of local safety policies, physical infrastructure improvements, and the improvement of child-safe environments.

## 6 Conclusion

This paper focuses on the important yet complex role of guardian-related factors in preventing unintentional injuries among rural children in China. Through complementary quantitative (Study I) and qualitative (Study II) approaches, we demonstrate that while guardianship significantly influences injury outcomes, effective prevention requires multi-level interventions addressing environmental, behavioral, and systemic factors.

We acknowledge several limitations: 1. Potential selection bias may be present in Study I, as some guardians (e.g., grandparents) may not use WeChat or may have declined to participate. This could result in an over- or under-estimation of certain associations. 2. As a cross-sectional design, Study I is limited in establishing causal relationships between guardian-related factors and injury outcomes. 3. In Study II, while we included diverse guardian and injury types, and conducted follow-up interviews to confirm saturation, we recognize that theme analysis may involve subjective interpretation and potential omissions.

The role of guardians in a child’s growth and development is undeniably crucial. Effective prevention of unintentional injuries in children must be grounded in enhanced guardian education and guardianship. Findings from Study I show that while guardian behavior and preventive practices can reduce the risk of injury to some extent, few factors also showed statistically significant effects, suggesting that unintentional injuries among rural children are influenced by a broader set of variables.

Building upon the Haddon Matrix framework, Study II offers a more comprehensive view of injury causation and prevention. It emphasizes that guardians must take proactive steps before an injury occurs, such as minimizing environmental risk factors and paying attention to children’s activities to reduce injuries caused by neglect or oversight.

During the injury event, guardians must respond quickly and effectively to interrupt the process and mitigate its severity. After an injury has occurred, it is essential to seek immediate medical attention, handle the child’s treatment appropriately, and adopt follow-up measures to prevent recurrence.

Ultimately, injury prevention should not rest solely on the shoulders of guardians. A collaborative effort involving families, local government, schools, and community institutions is essential. Guardians must actively participate in joint efforts that reflect real-world rural conditions, ensuring that education, supervision, and coordinated interventions are well-integrated and sustained. Only through such multi-level, community-based strategies can the incidence of childhood unintentional injuries be effectively reduced.

## Data Availability

The original contributions presented in the study are included in the article/supplementary material, further inquiries can be directed to the corresponding author.
